# Precutting endoscopic band ligation-assisted resection versus endoscopic submucosal dissection in patients with small gastric submucosal tumors originating from the muscularis propria: study protocol of a randomized controlled trial

**DOI:** 10.1186/s13063-024-07902-7

**Published:** 2024-01-13

**Authors:** Mingfeng Liu, Rui Yuan, Ke Zhan, Yichun Yang, Shan Li, Liang Deng

**Affiliations:** Department of Gastroenterology, The First Affiliated Hospital of Chongqing Medical University, Chongqing, China

**Keywords:** SMTs, SMT-MPs, GISTs, Precutting EBLR, ESD, Randomized controlled trial

## Abstract

**Background:**

The management of small gastric submucosal tumors (SMTs) originating from the muscularis propria layer (SMT-MPs) remains a subject of debate. Endoscopic submucosal dissection (ESD) is currently considered the optimal treatment for resection. However, high expenses, complex procedures, and the risk of complications have limited its application. Our previously proposed novel operation, precutting endoscopic band ligation (precutting EBL), has been demonstrated in a long-term, single-arm study to be an effective and safe technique for removing small gastric SMTs. However, the absence of a pathological examination and the potential for delayed perforation have raised concerns. Thus, we modified the precutting EBL by adding endoscopic resection to the snare after ligation and closure, yielding the precutting endoscopic band ligation-assisted resection (precutting EBLR). Moreover, the initial pilot study confirmed the safety and efficacy of the proposed approach and we planned a randomized controlled trial (RCT) to further validate its clinical feasibility.

**Methods:**

This was a prospective, single-center, open-label, parallel group, and randomized controlled trial. Approximately 40 patients with SMT-MPs will be included in this trial. The patients included were allocated to two groups: ESD and precutting EBLR. The basic clinical data of the patients were collected in detail. To better quantify the difference between ESD and precutting EBLR, the primary outcome was set as the operation duration. The secondary outcomes included total operation cost and hospitalization, intraoperative adverse events, and postoperative recurrence. The primary outcome was tested for superiority, while the secondary outcomes were tested for noninferiority. SPSS is commonly used for statistical analysis.

**Discussion:**

This study was designed to validate the feasibility of a novel operation for removing gastric SMT-MPs. To intuitively assess this phenomenon, the operation durations of precutting EBLR and ESD were compared, and other outcomes were also recorded comprehensively.

**Trial registration:**

Chinese Clinical Trial Registry ChiCTR2200065473. Registered on November 5, 2022.

**Supplementary Information:**

The online version contains supplementary material available at 10.1186/s13063-024-07902-7.

## Introduction

### Background and rationale

Submucosal tumors (SMTs) histologically include both epithelial and nonepithelial tumors. Nonepithelial tumors typically present as protruding lesions or masses covered with intact mucosa [1]. Large SMTs (≥2 cm) in the stomach may lead to early-stage complications such as bleeding or perforation, resulting in symptoms such as abdominal bloating, pain, hematemesis, or melena, which prompt patients to seek medical attention. In contrast, small gastric SMTs (<2 cm) are typically discovered incidentally during endoscopy without any apparent symptoms [2, 3].

The risk of small gastric SMT-MPs has been underestimated [4]. Studies suggest that 60–70% of SMT-MPs are pathologically identified as gastrointestinal stromal tumors (GISTs) and categorized as potential malignancies regardless of their size [2, 3]. However, although surgeons propose resection for large gastric SMT-MPs, clinical controversy persists [1, 5, 6]. In a retrospective study conducted by Ge QC et al. [7], a cutoff value of 1.48 cm was established to predict the malignant potential of GISTs. Tumors larger than 1.48 cm were associated with greater malignant potential, warranting intensive surveillance or endoscopic surgery. According to the modified National Institute of Health, the risk of small GISTs varies only with the mitotic count. The classifications included very-low risk (mitotic count ≤5), intermediate risk (mitotic count between 5 and 10), and high risk (mitotic count >10). Some advocate for imaging surveillance as the primary approach, suggesting resection only when tumor progression is confirmed. This includes cases where the tumor shows signs of increasing size, irregular borders, or pathological confirmation as a cancer [8]. Although endoscopic ultrasound (EUS) is a common method for diagnosing gastrointestinal superficial lesions, its role in diagnosing SMT-MPs has not been determined. Additionally, consistent observation of dynamic changes in tumor size and border length for patients with SMT-MPs < 16 mm is challenging. Although EUS-guided fine needle aspiration (EUS-FNA) is often employed for pathology, it may not fully reveal the pathological features of GISTs due to heterogeneity. In conclusion, en bloc resection is crucial for both diagnosis and prognosis [9].

Endoscopic resection, in comparison to open or laparoscopic surgery, yields a shorter operation duration, reduced blood loss, and shorter average hospitalization duration [9–14]. Endoscopic submucosal resection (ESD) has been demonstrated to be feasible for treating gastric SMTs. Guidelines from the European Society of Gastrointestinal Endoscopy (ESGE) and the American Society for Gastrointestinal Endoscopy (ASGE) recommend ESD as the preferred treatment for most gastric superficial neoplastic lesions [15, 16]. However, its effectiveness is limited for lesions originating from deeper layers such as the muscularis propria, increasing the complexity of the operation and the risk of complications. A systematic review by Ichiro Oda et al., encompassing more than 300 patients with early gastric cancer treated with ESD, identified several complications associated with the procedure. These complications included perforation (1.2–5.2%), bleeding (7% for immediate bleeding, up to 15.6% for delayed bleeding), stenosis (0.7–1.9%), aspiration pneumonia (0.8–1.6%), and air embolism, among others [17]. Although management strategies exist for these adverse events, they demand a higher level of technical expertise, adding to the financial burden and psychological stress on patients. Furthermore, ESD may not always achieve R0 resection, posing challenges for diagnosis and prognosis [18, 19]. According to an analysis of 733 patients with upper gastrointestinal SMT-MPs, extensive tumor connection was identified as a risk factor for incomplete resection [20]. In a multicenter prospective study by Ye LP et al. involving 692 patients, the R0 resection rate was 84.2% [19]. Hence, a more judicious treatment approach is imperative.

We previously introduced a novel endoscopic treatment termed precutting EBL. In this operation, an electrosurgical snare resection is performed to initially remove the mucosa surrounding the tumor, followed by the use of a transparent ligator to suction the tumor. A long-term, single-center study has substantiated its safety and efficacy. Precutting EBL was associated with a significantly shorter operation duration (16.6 min) and lower cost ($603.3 ± 5.9) than ESD ($2783 ± 601), and it was associated with fewer complications [21]. However, precutting EBL has two notable drawbacks. First, pathological specimens were not collected since the tumor spontaneously drops off after ligation, necessitating long-term follow-up for eradication verification. Second, like other ligate-and-let-go techniques, there is a risk of delayed perforation after the operation, which warrants careful consideration [22]. Given that we did not have sufficient samples to assess the possibility of delayed perforation, we opted to perform en bloc resection of lesions after ligation. Although this approach increases the chances of intraoperative perforation, we can promptly address this possibility if it occurs. Consequently, we propose a modified endoscopic operation for small gastric SMT-MPs, termed precutting EBLR. This involves an additional snare resection immediately after ligation. After thorough communication and detailed informed consent, we experimentally performed precutting EBLR on 16 patients. All patients showed rapid postoperative recovery, with no instances of delayed gastric bleeding or perforation. Importantly, subsequent pathological examination confirmed R0 resection in every patient.

To further enhance the clinical validation of precutting EBLR, we opted to initiate a randomized controlled trial comparing the efficacy and safety of ESD and precutting EBLR for the treatment of small gastric SMT-MPs.

### Trial design and objective

This was a single-center, open-label, parallel-group, randomized controlled trial. The main objective of this trial was to verify the efficacy and safety of precutting EBLR in the management of small gastric SMT-MPs. The trial began on December 1, 2022. The procedures included recruitment, informed consent, allocation of participants, intervention, data collection, data monitoring, and statistical analysis. All procedures were conducted at The First Affiliated Hospital of Chongqing Medical University (CQMU). A detailed flowchart for this trial is available in the Supplementary Materials. The drafting of this manuscript adheres to the SPIRIT reporting guidelines [23]. The SPIRIT checklist is attached as Additional file 2 in Supplementary Materials.

## Methods

### Definition

Several key definitions are outlined below:

1. Efficacy: the efficacy was determined based on the operation duration, operation cost, and hospitalization duration
2. Operation cost: the sum of the operational and material expenses, retrievable from the hospital system
3. Operation duration: the time from the administration of preoperative anesthesia to the patient's recovery of consciousness in the postoperative period
4. Safety: the ratio of intraoperative to postoperative complications
5. En bloc resection: complete removal of a lesion without any segmentation or partial lesion remaining
6. R0 resection: the absence of cancerous tissue on the edges of the lesion after resection
7. Postoperative gastric bleeding: a patient experienced hematemesis, melena, or an unexplained decrease in hemoglobin levels after the operation
8. Delayed perforation: the occurrence of sudden abdominal pain after the operation, accompanied by the detection of retroperitoneal pneumatosis or free gas through imaging examination
9. Postoperative recurrence: the discovery of a newly investigated tumor-like lesion that is eventually proven to be the same pathology as the previously resected tumor
10. Hospitalization duration: the number of days from admission to discharge

### Patient and public involvement

No patients or members of the public were involved in any way in the design of this trial.

### Recruitment

Patients with SMT-MPs admitted to The First Affiliated Hospital of CQMU were recruited. The inclusion criteria were as follows:

1. Age between 18 and 80 years
2. SMT-MPs with a diameter less than 1.6 cm confirmed through EUS
3. Preoperative computed tomography (CT) indicated no evidence of tumor metastasis in the liver or other organs
4. Willingness of the patient to undergo treatment with either ESD or precutting EBLR
5. Informed consent was obtained

The exclusion criteria were as follows:

1. EUS data were not available from The First Affiliated Hospital of CQMU or any other hospitals
2. Contraindications for gastroscopy or endoscopic surgery, such as cardiopulmonary insufficiency rendering the patient unsuitable for endoscopy, shock, or gastrointestinal perforation; inability to cooperate due to psychiatric disorders; acute severe laryngopharyngeal disorders preventing endoscope insertion; acute stage of corrosive esophageal injury; coagulation disorders; or a hemorrhagic tendency
3. Pregnant or breastfeeding
4. Presence of advanced malignant tumors
5. Allergy to oral lidocaine syrup and dimethicone oil
6. Current participation in other clinical trials
7. Option to withdraw from the trial exercised at any time

The entire recruitment process is managed by postgraduates MfL and RY. All patients with SMT-MP who met the inclusion criteria were approached for potential participation. Despite the absence of specific literature and sample data on enrollment and recruitment rates, achieving the desired sample size is deemed feasible based on the current participant flow. As the principal investigator of this trial, Physician LD assumes the responsibility of conducting comprehensive communication and obtaining informed consent from patients. Each participant received a copy of the informed consent form detailing the trial's potential benefits and risks. After thoughtful consideration, participants are empowered to make independent decisions about their involvement. The recruitment and informed consent process is devoid of inducements or pressures, ensuring voluntary participation and preventing unwarranted termination or loss to follow-up. Participants retain the option to withdraw from the trial at any point.

### Allocation

The sample size was determined based on the primary outcome, operation duration, using PASS 2011 software (NCSS, LLC, Kaysville, Utah, USA). Drawing from insights obtained from our previous single-arm retrospective study and a trial investigating ESD [24], with a power of 90% (*β* = 0.1) and a significance level (*α*) of 0.05 [25], the estimated primary sample size was approximately 34 patients. To account for a potential dropout rate of 10–20%, the final sample size was set at 40 patients. Consequently, each group included 20 patients.

Randomization was performed by SL using a random numbers table generated by IBM SPSS Statistics 23. From 1 to 40, each order was randomly assigned either the letter A or B with an equal probability. After the generation was completed, participants with the letter A were assigned to the Precutting EBLR group, while those with the letter B were assigned to the ESD group.

### Interventions

The participating operators were required to meet the following criteria:

1. Possess more than 5 years of experience in medicine
2. Demonstrated ability to independently conduct endoscopic operations
3. Operators with a history of performing no fewer than 300 endoscopic operations annually and a total of at least 1000 procedures

Patients were required to undergo a comprehensive preoperative evaluation to ensure the absence of absolute surgical contraindications. The operation was immediately stopped in the event of an unexpected intraoperative contingency, and appropriate clinical measures were taken accordingly. A detailed analysis and documentation of the possible reasons for such contingencies will be conducted. Intraoperative and postoperative interventions may be adjusted following established guidelines [26]. Implementing ESD or precutting EBLR will not require alteration to usual care pathways (including the use of any medication), and these steps will continue for both trial arms. Regarding postdischarge interventions (regular intake of esomeprazole), we contacted each participant via phone to provide reminders for consistency in medication adherence. This approach was approved by the participants when they signed the informed consent form.

#### ESD

Initially, a high-viscosity solution is employed to elevate the submucosal covering of the tumor. Subsequently, electrocautery knives are used for dissecting the tissue beneath and surrounding the lesion, leaving a resection bed. In the event of a perforation, closure can be facilitated using titanium clips or a purse-string suture [18]. Following the completion of the operation, patients undergo a 48-h observation period during which they fast and regularly take esomeprazole (40 mg, twice daily). Upon discharge, patients are required to continue taking esomeprazole (40 mg, once daily) for 2 weeks.

#### Precutting EBLR

**Fig. 1:**
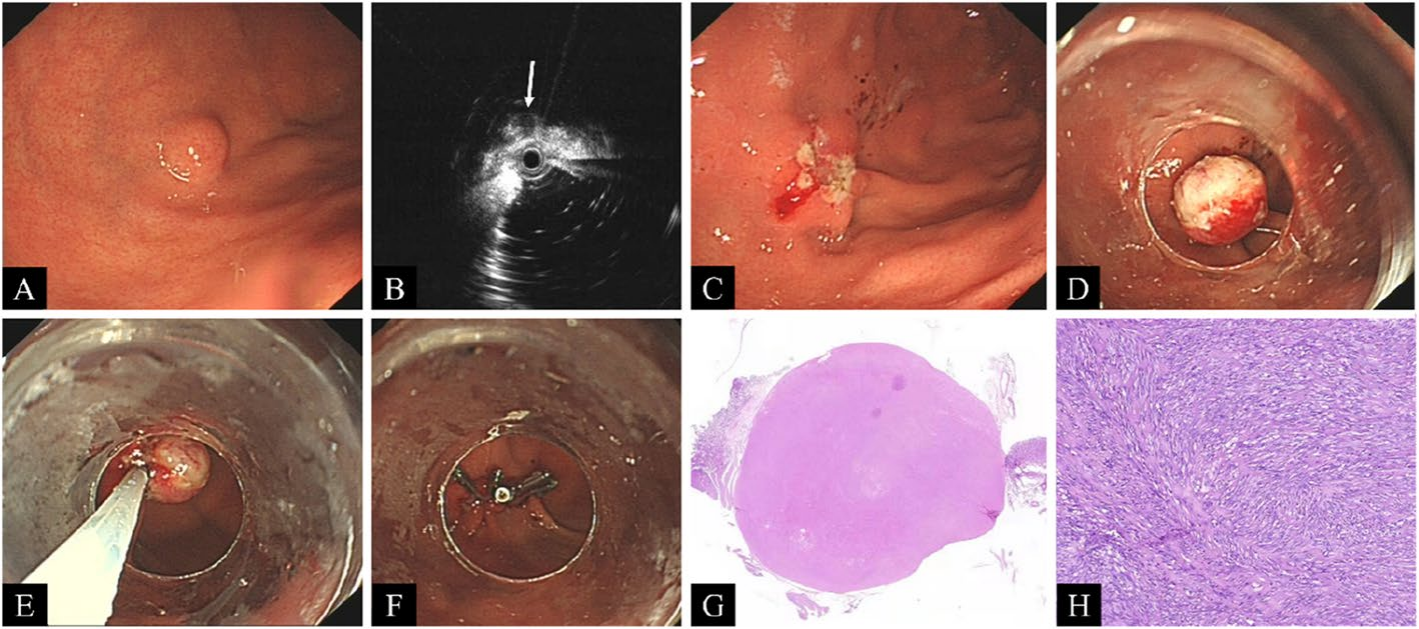
Steps of the precutting EBLR and postoperative pathology. **A** The tumor investigated in the fundus of the stomach by white light gastroscopy. **B** The tumor (white arrow) investigated by EUS. **C** The tumor’s covering mucosa was precut and removed. **D** The tumor was drawn from the surface using a transparent ligator. **E** The tumor was removed using an electrosurgical snare. **F** Active perforation was closed with titanium clips. **G** Microscopic view of the tumor. **H** Gastrointestinal stromal tumor was pathologically demonstrated

Initially, an electrosurgical snare is positioned on the tumor’s mucosal protuberance, followed by snare resection using an electrosurgical current set at 30 W to precut and remove the covering mucosa. Subsequently, an appropriate ligator is chosen based on the tumor size: a small ligator for tumors within 1 cm, a medium ligator for tumors ranging from 1 to 1.2 cm, and a large ligator for tumors greater than 1.2 cm. After proper ligator installation, the tumor is drawn from the surface and effectively removed using an electrosurgical snare. Closure of the perforation caused by ligation is assisted by employing three-armed clips or titanium clips. Finally, the excised tumor is sent for pathological examination. Postsurgery, fast for 12–24 h is required, followed by a liquid diet for 2–3 days and esomeprazole (40 mg, once daily) for 2 weeks. The steps of the operation and postoperative pathology are shown in Fig. 1*.*

### Devices

CT, EUS (OLYMPUS EU-M2000, 20 MHz, Japan), and standard endoscopy (AOHUA AQ200L, China) were used for preoperative assessment and follow-up. Standard endoscopes (AOHUA AQ200L, China) and loop snares (MICRO-TECH (NANJING) Co., Ltd., China) were used for mucosal protuberance precutting. Small ligators (TIANJIN TY, Medical Organism Material Research Company Ltd., China) were used for tumors ≤ 10 mm in length; medium ligators (OTSC cap plus ligation band, Ovesco Endoscopy AG, Tubingen, Germany) were used for tumors > 10 mm but ≤12 mm in length; and large ligators (colonoscopy transparent cap plus ligation band, OLYMPUS, Japan) were used for tumors >12 mm in length. All the ligators were disposable. Injectors (OLYMPUS, Japan), an IT knife, a dual knife, and an electronic cutting device (EREB VIO 200S, Germany) were used for ESD.

### Outcomes and follow-up

The primary outcome for the trial was operation duration, and the secondary outcome was operation cost. Both outcomes will be assessed prior to discharge. Additional meaningful indicators, also set as secondary outcomes, include estimated blood loss, intraoperative and postoperative adverse events (such as bleeding, immediate and delayed perforation, infection), tumor recurrence, mortality rates, and hospitalization costs.

The trial’s endpoint will be established as 6 months after the operation of the last included patient. Each patient is given a detailed follow-up evaluation via telephone 6 months after discharge to gather information about their postoperative condition. At the 6-month mark, patients are required to undergo an endoscopic re-examination to assess tumor recurrence.

## Data management

### Collection

All the data were collected and verified by two statisticians simultaneously using a spreadsheet (Microsoft Excel 2016) in accordance with each patient’s personal information and medical images. Patients are assigned numerical codes instead of their names to ensure the confidentiality of personal information. To minimize statistical errors, any controversial data is reviewed and discussed by a third person. Preoperative, intraoperative, and postoperative data are collected. Missing data will be declared in the appendix, and the corresponding participant will be considered withdrawn.

Preoperative data included demographic information (age, sex, date of admission) and tumor characteristics (size, layer, location, shape, and density of the echo site investigated via EUS). Intraoperative data included the operation date, duration, estimated blood loss, and details related to intraoperative perforation (size, duration, and amount of titanium clips). Postoperative data included the size of the resected tumor (assessed by ruler), tumor pathology (tumor type, mitotic count, achievement of R0 resection or not, and immunohistochemistry), postoperative management (duration of fasting and liquid diet, use of medications), postoperative symptoms and adverse events, hospitalization duration and cost, operation cost, and 6-month follow-up outcome.

### Monitoring

The data were monitored by The First Affiliated Hospital of CQMU. In this trial, the platform is exclusively utilized for hospitalization purposes and remains independent of any competing interests. Monthly trial audits will be conducted without the presence of funders or sponsors to assess the progress of each participant. LD will conduct an interim analysis around June 2024, and the trial may be terminated earlier than planned if the data are sufficiently convincing to draw a final conclusion or if a significant proportion of precutting EBLR patients develop unexpected postoperative complications. Adverse events (AEs) or severe adverse events (SAEs) will be promptly reported to the clinical trial team. Relevant information will also be recorded locally for further analysis.

### Statistical analysis

For statistical analysis, commercial software, specifically IBM SPSS Statistics 23, will be used. Normally distributed data are presented as the means and standard deviations (X±S). Student’s *t* test was used to analyze significant differences between groups. The data that conformed to a skewed distribution are expressed as the median and range. Statistical differences between groups were analyzed using the Mann–Whitney *U* test. Categorical data are presented as numbers and percentages and were analyzed using Fisher’s exact test or the chi-square test. To explore potential risk factors, participants were allocated to two subgroups based on tumor recurrence. Relevant data, including age, operation duration, tumor size, tumor layer, pathology, and mitotic count (if there was a GIST), were collected again. Univariate analysis will be conducted to identify differential expression of the genes. Multiple regression analysis was subsequently conducted on the various indicators. *P* < 0.05 indicated statistical significance.

### Participant timeline

See Table 1.

**Table 1:** Participant timeline

	Study period						
	Recruitment	Allocation	Hospitalization			Follow-up	
Timepoint	*−t*_*1*_	*0*	*Admission*	*Operation*	*Discharge*	*6 months*	*12 months*
**Recruitment:**							
**Eligibility screen**	X						
**Informed consent**	X						
**Allocation**		X					
**Interventions:**							
**ESD**				X			
**Precutting EBLR**				X			
**Assessments:**							
**Baseline characteristics**			X				
**Operation duration**				X			
**Estimated blood loss**				X			
**Intraoperative adverse events**				X			
**Postoperative adverse events**					X	X	X
**Operation cost**				X			
**Hospitalization cost**					X		

## Discussion

Initially, precutting EBL was designed to address the current challenge of treating small gastric SMT-MPs, and a previous study demonstrated its clinical feasibility. However, before we could extend its application to other areas or institutions, notable shortcomings emerged. In response, we promptly started to further modify the operation. This is why precutting EBLs were not tested on a larger scale. Simultaneously, a case of delayed perforation heightened our concern. Forty-eight hours after receiving precutting EBL, a middle-aged male patient suddenly complained of severe abdominal pain. A CT scan revealed a gastric perforation at the site of the lesion. Fortunately, the patient soon recovered and was discharged after immediate closure of the perforation. This case indicated a way to further modify precutting EBL to a certain extent.

Precutting EBLR was proposed. Its local performance in 16 patients revealed its advantages in terms of a shorter operation duration and lower expenses. Encouraged by these findings, we decided to gradually expand the scale of the study in anticipation of promoting precutting EBLR. The current trial is specifically designed to compare precutting EBLR and ESD. The operations were conducted at The First Affiliated Hospital of CQMU, a large-scale 3A general teaching hospital renowned for its high level of clinical and academic research. Located in southwestern China, the hospital attracts a substantial amount of patient flow, mainly from the surrounding regions and provinces. The Department of Gastroenterology at this hospital handles an extensive patient population, both in terms of quantity and variety, providing the necessary conditions to achieve the planned sample size. Prior to conducting this RCT, we collected primary data from 16 patients who underwent precutting EBLR. The data showed a mean operation duration of 21.3 ± 4.5 min (Table 2). Moreover, we performed a retrospective study involving 537 patients in whom the use of endoscopic resection for the treatment of small gastric SMTs was analyzed. The study revealed a mean operation duration of 38.3 ± 21.8 min.^24^ The shorter operation duration of precutting EBLR was evident. In this RCT, we designated the operation duration as the primary outcome. Based on sample size estimation guidelines for clinical studies, we set α and β to 0.05 and 0.9, respectively. With the above numerical values, 17 patients were included in one group. In other words, 34 participants were necessary in total for a 1:1 group ratio. We considered a 10–20% drop-out rate. The final sample size was determined to be 40 patients in total.

**Table 2:** Perioperative outcomes and follow-up results of precutting EBLR on 16 patients

Category	
Tumor specimen characteristics	
Tumor specimen size^a^, cm	
Mean ± SD	1.1 ± 0.2
Median (range)	1.0 (0.8–1.4)
Pathological diagnosis, No.(%)	
GIST	11 (68.8%)
Leiomyoma	5 (31.2%)
Operative outcomes	
En bloc resection	16 (100%)
R0 resection	16 (100%)
Operative time, min	
Mean ± SD	21.3 ± 4.5
Median (range)	21.0 (14.0–30.0)
Intraoperative perforation, No.(%)	10 (68.8%)
Adverse events, No.(%)	
Major bleeding	0 (0%)
Delayed perforation	0 (0%)
Fever	0 (0%)
Peritonitis	0 (0%)
Operative cost, $	
Mean ± SD	588.6 ± 55.4
Median (range)	583.0 (499.0–710.0)
Follow-up outcomes	
Recurrence, No.(%)	0 (%)

^a^Specimens’ sizes were measured by rulers after resections

The primary outcome was set as the operation duration, with the objective of showcasing the main advantage of precutting EBLR. The other outcomes also help to demonstrate the safety and efficacy of the treatment, such as reduced hospitalization costs when the duration is equal or shorter hospitalization duration when the costs are equal. Precutting EBLR holds the potential to emerge as a creative and promising endoscopic approach for treating SMT-MPs, offering a more practical, simpler, and safer alternative. Moreover, this approach has the potential to alleviate the economic burden on both patients and health insurance companies, leading to substantial societal benefits. These advantages also foster the prospect of transforming the resection of gastric small SMT-MPs from a hospitalized operation to an ambulatory operation.

This trial has several limitations. The relatively small sample size may introduce bias if patients are lost to follow-up, and conducting further multicenter studies could address this issue. Additionally, the 6-month follow-up duration might be insufficient to thoroughly observe tumor recurrence. Currently, there is a lack of a specific method for investigating tumor recurrence in a timely manner. In other words, if tumor recurrence occurs at 1 or 6 months after the operation, it is ultimately identified during the re-examination 6 months after discharge. This may lead to an underestimation of the impact of different operations on tumor recurrence.

### Supplementary Information

**Additional file 1.**

**Additional file 2.**

## Data Availability

No identifying images or other personal or clinical details of the participants are presented here or will be presented in reports of the trial results. The datasets used and/or analyzed during the current study, the participant information materials and the informed consent form are available from the corresponding author upon request. The trial results will be available via publication.

## Abbreviations

SMT: Submucosal tumor
SMT-MP: Submucosal tumor originating from the muscularis propria layer
ESD: Endoscopic submucosal dissection
Precutting EBL: Precutting endoscopic ligation
Precutting EBLR: Precutting endoscopic band ligation-assisted resection
RCT: Randomized controlled trial
GIST: Gastrointestinal stromal tumor
EUS: Endoscopic ultrasound
CT: Computed tomography
EUS-FNA: EUS-guided fine needle aspiration
AE: Adverse event
SAE: Severe adverse event
