# Supplementary material for: Precutting endoscopic band ligation-assisted resection versus endoscopic submucosal dissection in patients with small gastric submucosal tumors originating from the muscularis propria: study protocol of a randomized controlled trial
Source: Trials. 2024 Jan 13;25:49. doi: 10.1186/s13063-024-07902-7 (PMC10788014; doi:10.1186/s13063-024-07902-7)
Supplement: Supplementary file 1 — Additional file 1. [file 13063_2024_7902_MOESM1_ESM.docx]

Enrollment

Randomized (n=40)

Assessed for eligibility (n= )

Excluded (n= )

‣ Not meeting the inclusion criteria (n= )

‣ Refused to participate (n= )

‣ Other reasons (n= )

Analysis

Modified intention to treat (n= )

As treated (n= )

Per protocol (n= )

Per protocol (n= )

As treated (n= )

Modified intention to treat (n= )

Follow-up

Analysis (n= )

‣ Excluded from analysis (record reasons) (n= )

Analysis (n= )

‣ Excluded from analysis (record reasons) (n= )

Allocation

6 months follow-up (n= )

‣ Lost to follow-up (record reasons) (n= )

6 months follow-up (n= )

‣ Lost to follow-up (record reasons) (n= )

Allocated to intervention ESD

‣ Received allocated intervention (n= )

‣ Did not receive allocated intervention (record reasons) (n= )

‣ ······

Allocated to intervention Precutting EBLR

‣ Received allocated intervention (n= )

‣ Did not receive allocated intervention (record reasons) (n= )

‣ ······
